# Towards engineering a hybrid carboxysome

**DOI:** 10.1007/s11120-023-01009-x

**Published:** 2023-03-09

**Authors:** Nghiem Dinh Nguyen, Sacha B. Pulsford, Wei Yi Hee, Benjamin D. Rae, Loraine M. Rourke, G. Dean Price, Benedict M. Long

**Affiliations:** 1 Australian Research Council Centre of Excellence for Translational Photosynthesis, Research School of Biology, The Australian National University, Building 134, Linnaeus Way, Acton, ACT 2601 Australia; 2 Australian Research Council Centre of Excellence in Synthetic Biology, Research School of Chemistry, The Australian National University, Building 46, Sullivan’s Creek Road, Acton, ACT 2601 Australia; 3 Realizing Increased Photosynthetic Efficiency (RIPE), The Australian National University, 134 Linnaeus Way, Acton, ACT 2601 Australia; 4 Division of Plant Sciences, Research School of Biology, The Australian National University, Building 134, Linnaeus Way, Canberra, ACT 2601 Australia

**Keywords:** Rubisco, Rubisco chaperone, Rubisco condensation, Carbonic anhydrase, Carboxysome, CO_2_-concentrating mechanism

## Abstract

Carboxysomes are bacterial microcompartments, whose structural features enable the encapsulated Rubisco holoenzyme to operate in a high-CO_2_ environment. Consequently, Rubiscos housed within these compartments possess higher catalytic turnover rates relative to their plant counterparts. This particular enzymatic property has made the carboxysome, along with associated transporters, an attractive prospect to incorporate into plant chloroplasts to increase future crop yields. To date, two carboxysome types have been characterized, the α-type that has fewer shell components and the β-type that houses a faster Rubisco. While research is underway to construct a native carboxysome *in planta*, work investigating the internal arrangement of carboxysomes has identified conserved Rubisco amino acid residues between the two carboxysome types which could be engineered to produce a new, hybrid carboxysome. In theory, this hybrid carboxysome would benefit from the simpler α-carboxysome shell architecture while simultaneously exploiting the higher Rubisco turnover rates in β-carboxysomes. Here, we demonstrate in an *Escherichia coli* expression system, that the *Thermosynechococcus elongatus* Form IB Rubisco can be imperfectly incorporated into simplified *Cyanobium* α-carboxysome-like structures. While encapsulation of non-native cargo can be achieved, *T. elongatus* Form IB Rubisco does not interact with the *Cyanobium* carbonic anhydrase, a core requirement for proper carboxysome functionality. Together, these results suggest a way forward to hybrid carboxysome formation.

**Supplementary Information:**

The online version contains supplementary material available at 10.1007/s11120-023-01009-x.

## Introduction

d-ribulose-1,5-bisphosphate carboxylase/oxygenase (Rubisco) is critical for the conversion of photosynthetic chemical energy into fixed organic carbon through the Calvin-Benson-Bassham (CBB) cycle—that is, fixation of atmospheric CO_2_ to produce reduced carbon compounds (Sharwood 2017). The folding of the Rubisco protein complex is dependent on various chaperones and chaperonins, while its activity is further dependent on ions and several protein cofactors such as activases (Hayer-Hartl et al. 2016; Bracher et al. 2017; Mueller-Cajar 2017). While this enzyme has a significant role in global photosynthesis (Bar-On and Milo 2019), in plants the enzyme has a relatively slow turnover rate and poor discrimination between CO_2_ and O_2_ leading to inefficient inorganic carbon (C_i_) fixation (Tcherkez et al. 2006; Hagemann and Bauwe 2016; Zhou and Whitney 2019). As a corollary of these inherent inefficiencies in the Rubisco reaction, a myriad of complex processes collectively termed CO_2_-concentrating mechanisms (CCMs) have evolved in disparate photosynthetic organisms (Raven et al. 2008). Some examples of CCMs include C_4_ plants, and feature pyrenoids in green algae and carboxysomes in some bacteria and all cyanobacteria.

The bacterial CCM has several defining features. Initially, HCO_3_^−^ is actively accumulated within the cytoplasm and maintained out of chemical equilibrium with CO_2_ (Price 2011; Rottet et al. 2021). This then diffuses into large proteinaceous bacterial microcompartments called carboxysomes which house Rubisco and the carbonic anhydrase enzyme (Rae et al. 2013). These structures are essential to the success of the CCM (Berry et al. 2005; Long et al. 2007), with the carbonic anhydrase rapidly converting HCO_3_^−^ to CO_2_ that then accumulates at the site of Rubisco. In this way, the CCM floods Rubisco with substrate, enhancing CO_2_ fixation rates. Carboxysomes are typically 90–400 nm in diameter, icosahedral in shape and contain most, if not all, of the cell’s Rubisco (Yeates et al. 2008; Rae et al. 2013; Kerfeld and Melnicki 2016; Liu 2022).

**Fig. 1:**
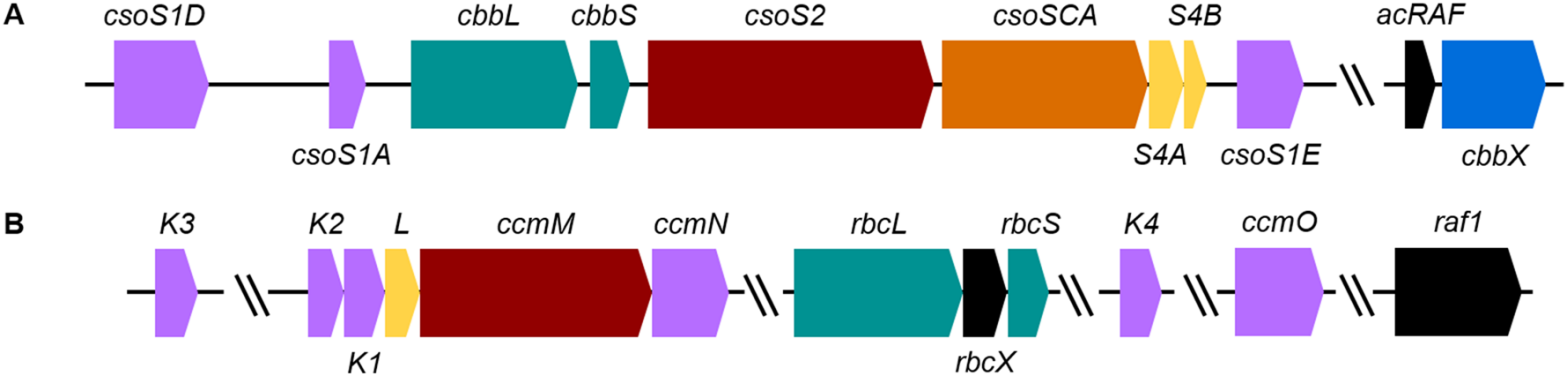
Carboxysome operon structures from **a** *Cyanobium* PCC7001 and **b** *T. elongatus* BP-1. Genes encoding proteins with conserved functions have the same colour, while doubled angled lines represent distant genomic regions. Genes coloured teal encode Rubisco, black genes encode Rubisco chaperones and blue genes encode Rubisco activases. Purple genes encode carboxysome shell proteins, yellow genes encode carboxysome vertex proteins, orange genes encode carbonic anhydrase enzyme and brown genes encode carboxysome Rubisco binding partners

Two independent classes of carboxysomes have emerged in cyanobacteria, the α-carboxysome which is of proteobacterial origin and contains the Form IA Rubisco found in both proteobacteria and α-cyanobacteria (Badger et al. 2002; Cabello-Yeves et al. 2022), and the β- which arose in β-cyanobacteria and houses the Form IB Rubisco homologous to that seen in terrestrial phototrophs (Badger et al. 2002; Cabello-Yeves et al. 2022). The α-carboxysome is encoded by the *cso* operon while the β-carboxysome is encoded by the *ccm* operon and gene clusters (Yeates et al. 2008; Rae et al. 2013; Kerfeld and Melnicki 2016; Liu 2022). These operons/gene clusters of α- and β-cyanobacteria are of distinct phylogenetic origin and contain genes encoding carboxysomal shell and scaffold proteins, either cyanobacterial Form IA Rubisco (*cbbLS*) or Form IB Rubisco (*rbcLS*), specific Rubisco chaperone proteins and carbonic anhydrases (Fig. 1, Badger and Bek 2008).

Despite these differences in componentry and origin, there are some similarities in the biogenesis pathway of these divergent carboxysomes. Across the two types, Rubisco must interact with its cognate carboxysome binding partner (CsoS2 in α-carboxysomes Cai et al. 2015; Chaijarasphong et al. 2016; Oltrogge et al. 2020); CcmM in β-carboxysomes (Long et al. 2007, 2010, 2011; Cot et al. 2008; Ryan et al. 2019; Wang et al. 2019; Zang et al. 2021)) before its encapsulation by carboxysome shell proteins. Both the Form IA Rubisco:CsoS2 and the Form IB Rubisco:CcmM interactions are now known to occur at the equatorial region of the Rubisco holoenzyme and facilitate liquid–liquid phase separation (Wang et al. 2019; Oltrogge et al. 2020; Zang et al. 2021). The similarity in binding interfaces between Rubisco and the two carboxysome binding partners CsoS2 and CcmM, suggests that the Rubisco forms have the potential to interact with both CsoS2 and CcmM. This would be the first requisite step to constructing a new, hybrid carboxysome containing proteins from both the α- and β-carboxysome.

What would be the benefit of a hybrid carboxysome? While the concept of a hybrid carboxysome may be of academic interest, it is worthwhile rationalizing this idea to best understand how this system may prove more beneficial than a native carboxysomal system in our attempts to improve crop yields using cyanobacterial components (Parry et al. 2011; Rae et al. 2017; Hennacy and Jonikas 2020). Form IB Rubiscos found in β-carboxysomes tend to have higher catalytic rates relative to their Form IA counterparts that are found in α-carboxysomes (Whitehead et al. 2014; Flamholz et al. 2019). On the other hand, α-carboxysomes are simpler to assemble with fewer components required to produce a shell structure, with only CsoS1A and CsoS2 required for the assembly of a simple α-carboxysome (Long et al. 2018). Comparatively, the β-carboxysome shell has more components (Sommer et al. 2017, 2019), with the simplest β-carboxysome requiring the co-expression of CcmK2, CcmO, CcmM and CcmL (Occhialini et al. 2016).

With these defining characteristics in mind, it logically follows that if the interaction between Rubisco and its carboxysomal binding partner can be resolved, then a Form IB Rubisco can be encapsulated by an α-carboxysome shell to generate a hybrid carboxysome that may be simple to form and in theory, may outperform its native counterpart, in net carboxylation rates.

This study examined whether the Form IB Rubisco from *T. elongatus* BP-1 (hereafter *T. elongatus*) could be successfully encapsulated within a simplified *Cyanobium* PCC7001 (hereafter *Cyanobium*) α-carboxysome. The catalytic turnover rate of the *T. elongatus* Rubisco used here is not as rapid as that of the fastest carboxysomal Form IB enzyme (Whitehead et al. 2014; Flamholz et al. 2019; Zhou and Whitney 2019). However, it’s sequence and surface property similarity with other carboxysomal Form IB Rubiscos make it a good proof of concept for the potential of Form IB Rubisco encapsulation within an α-carboxysome. To address this aim, site-specific mutation was employed to generate an α-carboxysome-compatible Form IB Rubisco by introducing specific surface-interface residues responsible for the CsoS2:Rubisco interaction normally found in α-carboxysomes. Modified *T. elongatus* Rubisco was then assessed for holoenzyme assembly, chaperone requirements, catalytic turnover rates and carboxysome encapsulation. The interaction between *T. elongatus* Rubisco and *Cyanobium* CsoSCA was also assessed.

## Results

### The Rubisco:CsoS2 interface is conserved across both α- and β-cyanobacterial Rubiscos

**Fig. 2:**
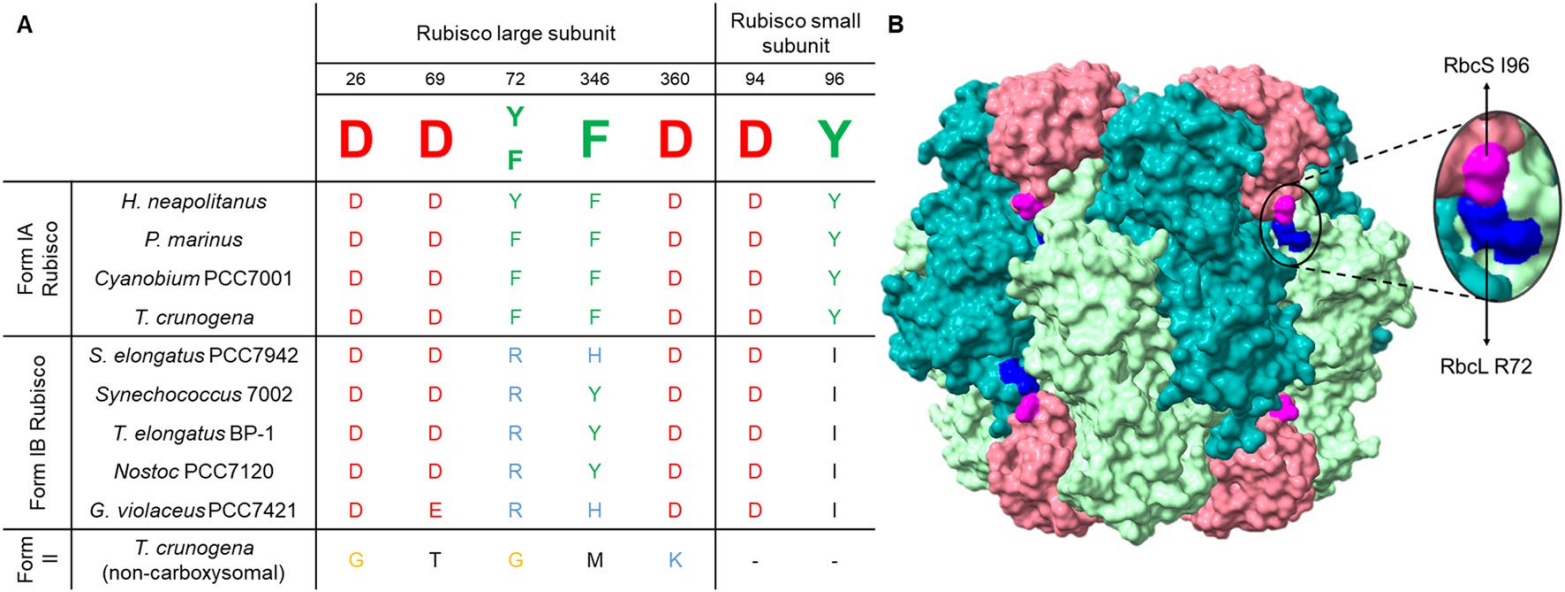
**a** Rubisco residues responsible for the Rubisco-CsoS2 interaction are widely conserved across both Form IA and IB Rubiscos and **b** proposed mutations in *T. elongatus* Rubisco to improve its ability to interact with *Cyanobium* PCC7001 CsoS2. Comparison of CsoS2 interacting amino acid residues across Form IA and Form IB Rubisco sequences with a consensus sequence from an alignment of Form IA Rubisco sequences, adapted from Oltrogge et al. (2020). Residues are numbered according to the *H. neapolitanus* Rubisco large and small subunit sequence. The residues critical for the Rubisco-CsoS2 interaction were mostly conserved across the Form IA Rubiscos examined. Form IB Rubiscos were also found to contain many of the critical residues for the Rubisco-CsoS2 interaction, with *T. elongatus* possessing four such residues. Surface structure of *T. elongatus* Rubisco (PDB:2YBV) showing the variable amino acids compared with the Form IA Rubisco:CsoS2 interacting domain identified by Oltrogge et al. (2020)

Using the identified Form IA Rubisco:CsoS2 interacting amino acid residues as a guide (Oltrogge et al. 2020) we aligned the Rubisco large and small subunits protein sequences from key species of interest (Fig. 2a) to identify candidate residues for mutagenesis. The critical Rubisco residues responsible for the Rubisco:CsoS2 interaction were largely conserved across the Form IA Rubiscos (Fig. 2a; Oltrogge et al. 2020). Form IB Rubiscos contain many of the Rubisco:CsoS2 interacting residues as identified from the Form IA Rubiscos, with *T. elongatus* possessing four such residues (Fig. 2a). The non-carboxysome Form II Rubisco from *T. crunogena* lacked all the Rubisco:CsoS2 interacting residues (Fig. 2a). The conserved nature of these residues suggests that it is not beyond reason for Form IB Rubisco to interact with CsoS2, and consequently be encapsulated within the α-carboxysome. We therefore hypothesised that two mutations could be introduced into *T. elongatus* Rubisco, namely R72F in *T. elongatus* RbcL and I96Y in *T. elongatus* RbcS (Fig. 2a) to enable its encapsulation within an α-carboxysome shell. Structurally both residues reside in the Rubisco equatorial region as highlighted in Fig. 2b. We consider here that a Y346F mutation in *T. elongatus* RbcL does not represent as critical a residue change as those expected for R72F and RbcS I96Y, although it should be noted that the hydroxyl of Y346 may contribute to interactions.

### Mutant *T. elongatus* Rubiscos can still undergo chaperone-assisted oligomerisation and are catalytically active

**Fig. 3:**
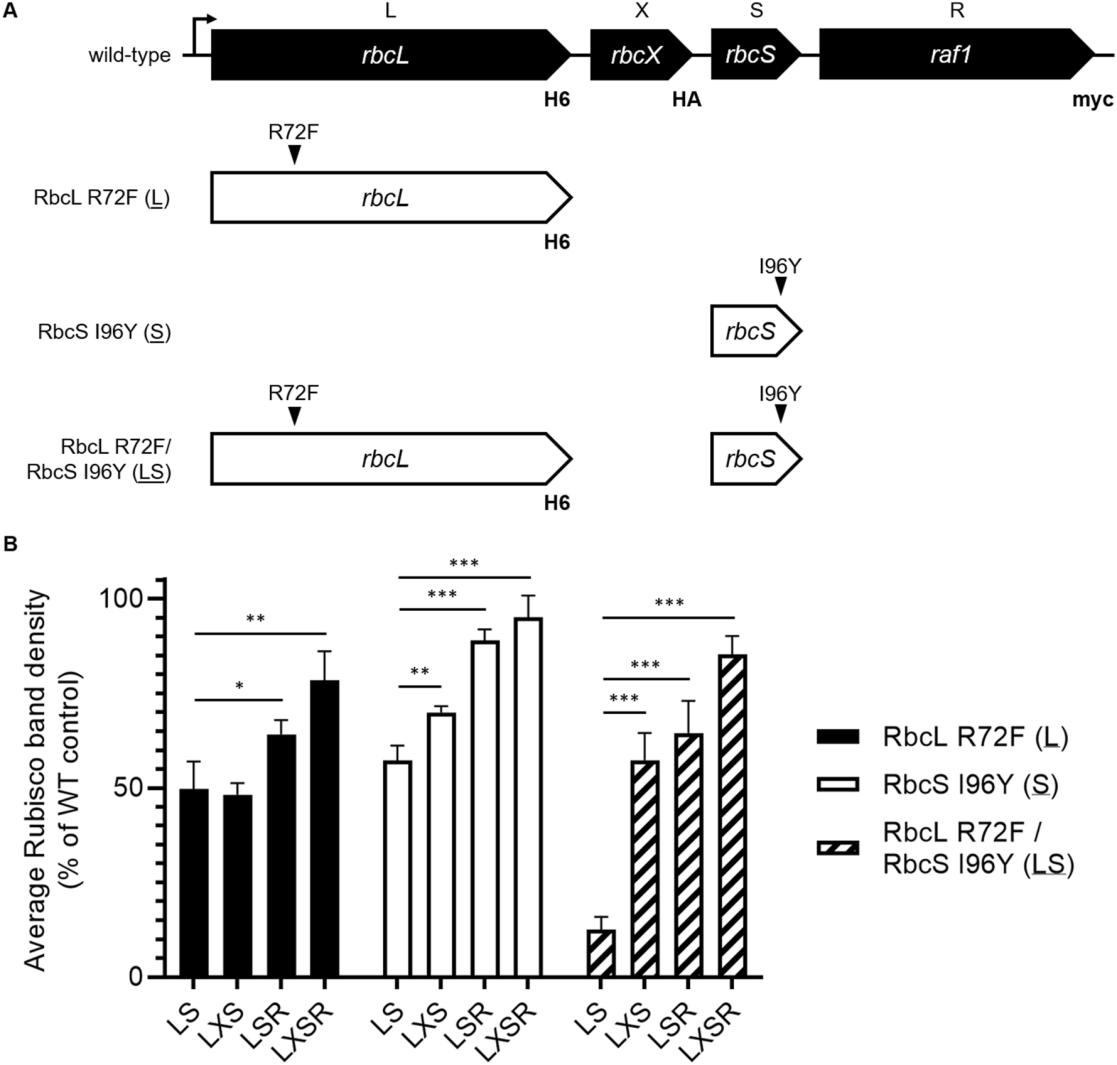
**a** Schematic of constructs used to determine if the mutated *T. elongatus* Rubisco had different chaperone requirements, or if these mutated Rubiscos had different kinetic parameters. **b** *T. elongatus* Rubisco mutants (RbcL R72F, RbcS I96Y and RbcL R72F/RbcS I96Y) require Rubisco chaperone proteins RbcX and Raf1 for maximum Rubisco holoenzyme assembly. Here, *rbcL* was synthesised with a 6 × His-tag (HHHHHH), *rbcX* was synthesised with a HA-tag (YPYDVPDYA) and *raf1* was synthesised with a myc-tag (EQKLISEEDL) to facilitate immunoblot detection. Rubisco holoenzyme band densities were determined from Coomassie stained native gels of soluble proteins from *E. coli* cells expressing *T. elongatus* Rubisco mutants (RbcL R72F, RbcS I96Y and RbcL R72F/RbcS I96Y) without any chaperones (LS), with just RbcX (LXS) or Raf1 (LSR) and with both RbcX and Raf1 (LXSR). Band densities were quantified using Imagelab software (Bio-Rad) and compared across gels using an internal control (wild-type *T. elongatus* Rubisco co-expressed with RbcX and Raf1). For all three Rubisco mutants (RbcL R72F, RbcS I96Y and RbcL R72F/RbcS I96Y) co-expression with both RbcX and Raf1 resulted in a maximum Rubisco formation (*p* < 0.01; *p* < 0.001). Data are an average of three biological replicates and asterisks denote significant difference from Rubisco mutant expressed on its own (*p < 0.05; **p < 0.01; ***p < 0.001; Tukey’s Post Hoc Test)

The successful folding and assembly of cyanobacterial Form IB Rubisco is reliant upon the chaperones RbcX and Raf1 (Hauser et al. 2015). Since the modification of *T. elongatus* Rubisco surface residues to match those of a Form IA enzyme may also affect chaperone interactions, we also evaluated the ability of RbcX and Raf1 to assemble mutated enzymes (Figure S1). Mutations introduced into *T. elongatus* Rubisco (RbcL R72F and RbcS I96Y) did not prevent *T. elongatus* Rubisco holoenzyme assembly if chaperones were co-expressed (Figs. 3, S2 and S3). Moreover, both chaperones were required to produce the highest Rubisco yield (Figs. 3, S2).

Catalytically, Form IB Rubiscos are generally reported to have faster carboxylation rates than their Form IA counterparts, for example, *Synechococcus elongatus* PCC7942 Rubisco has a catalytic turnover rate (*k*_cat_) of 14 s^−1^ while *Cyanobium* Rubisco has a *k*_cat_ value of 9 s^−1^ (Whitehead et al. 2014; Flamholz et al. 2019). This served as a motivating factor for generating a hybrid carboxysome in this study. In generating a mutant *T. elongatus* Rubisco, it was unknown if the enzyme’s catalytic properties would still be maintained. To assess this, single point Rubisco turnover assays were performed to estimate catalytic activity for each mutant enzyme. The addition of the His-tag on the C-terminus of *T. elongatus* Rubisco significantly decreased the holoenzyme’s *k*_cat_ (*p* < 0.05). However, the mutations introduced into *T. elongatus* Rubisco did not significantly affect enzyme turnover (Table 1).

**Table 1:** Carboxylation turnover rates *k*_(cat)_ (s^−1^) values for wild-type and mutant *T. elongatus* Rubisco enzymes at 25 °C

*T. elongatus* Rubisco type	*k*_(cat)_ (s^−1^)
WT *T. elongatus* Rubisco	4.04 ± 0.44
His tagged—*T. elongatus* Rubisco	3.02 ± 0.35*
His tagged—*T. elongatus* Rubisco, RbcL R72F	3.12 ± 0.47
His tagged—*T. elongatus* Rubisco, RbcL R72F and RbcS I96Y	3.16 ± 0.19

Rubisco ^14^CO_2_ carboxylation and ^14^C-CABP binding assays were performed. The addition of the His-tag on the C-terminus of *T. elongatus* resulted in a statistically significant difference in the holoenzyme’s *k*_(cat)_ (one-way ANOVA, * *p* < 0.05), however, the introduction of mutations into *T. elongatus* RbcL and RbcS did not confer any significant difference to this catalytic parameter. Data represented as means ± s.d. of three biological replicates, each represented by three technical replicates

### Wild-type *T. elongatus* Form IB Rubisco can be encapsulated within a simplified *Cyanobium* carboxysome

**Fig. 4:**
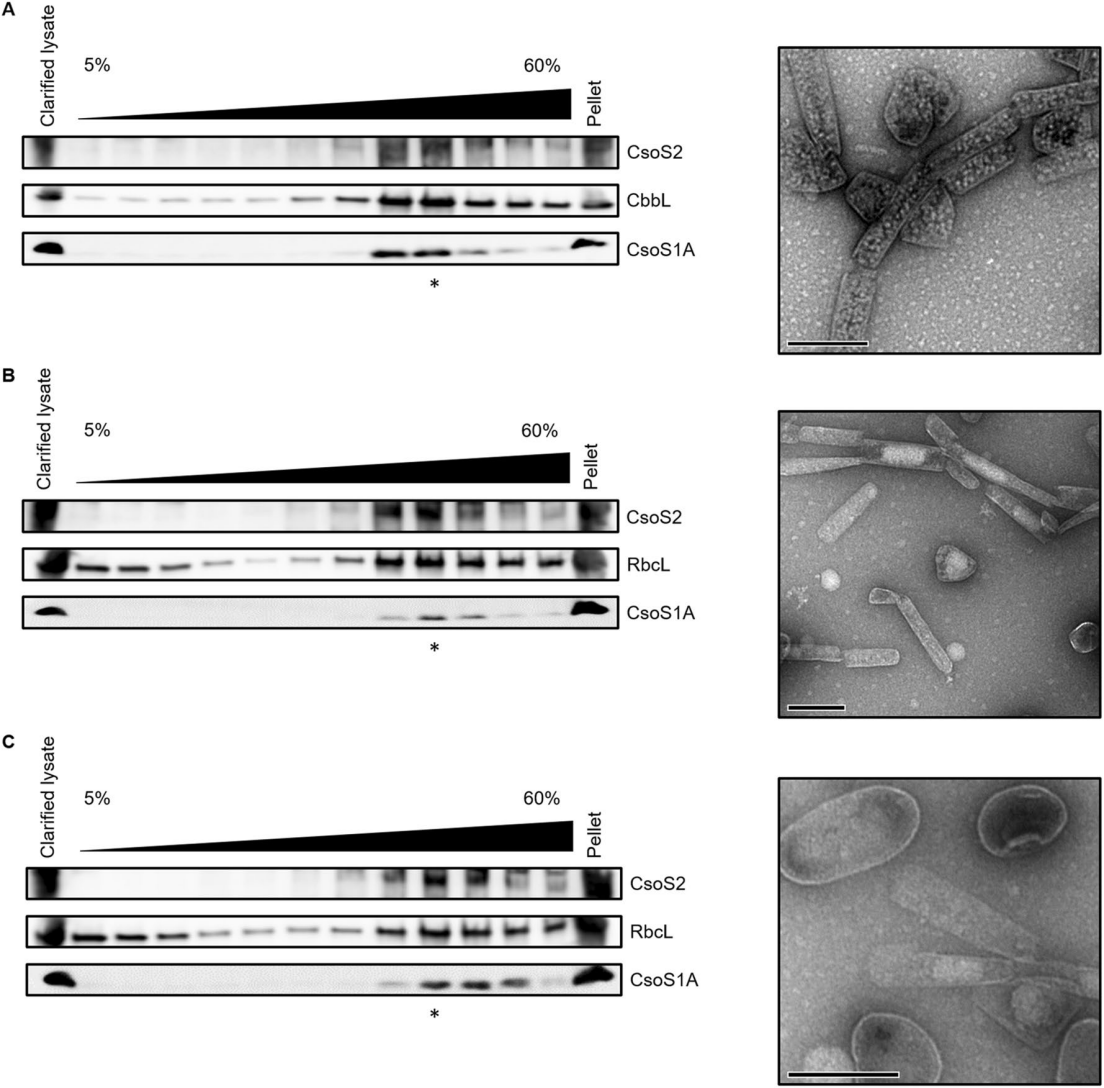
Simplified *Cyanobium* PCC7001 carboxysome structures (**a**) and rod-like carboxysome structures produced from the co-expression of wild-type (**b**) or mutant (**c**) *T. elongatus* Rubisco and *Cyanobium* PCC7001 CsoS1A and CsoS2, can be purified on sucrose gradients. Western blots were used to determine the sucrose gradient fraction that contained the greatest quantity of proteins (Rubisco, CsoS1A and CsoS2) which were observed by TEM (indicated by the asterisk). The co-expression of *Cyanobium* PCC7001 Rubisco, CsoS1A and CsoS2 produced rod-like carboxysome structures containing sub-structure reminiscent of holoenzymes (**a**). The co-expression of wild-type (**b**) and modified (**c**) *T. elongatus* Rubisco and *Cyanobium* PCC7001 carboxysomes components (CsoS1A and CsoS2) produced rod-like carboxysome structures with inconsistent internal structure. Scale bar represents 200 nm

We next sought to assess the encapsulation of different Rubiscos with the simplified *Cyanobium* α-carboxysome system. Sucrose gradient fractionation on *E. coli* cell lysates from cell lines expressing carboxysome components was performed to separate free Rubisco holoenzymes from encapsulated Rubisco (Fig. 4).

Control preparations from *E. coli* cells expressing the simplified *Cyanobium* carboxysome and its unmodified Form IA Rubisco (Figure S1b) show a clear Rubisco peak towards the lower section of the gradient that coalesced with CsoS1A and CsoS2 (Figs. 4a, S4a). The co-localisation of *Cyanobium* CsoS1A, CsoS2 and Rubisco in dense sucrose gradient fractions is consistent with the successful encapsulation of Rubisco in these heterologously expressed structures. Trace amounts of *Cyanobium* Rubisco were also detected at the top of the sucrose gradient, indicative of some free Rubisco in these preparations (Fig. 4a). Transmission electron microscopy (TEM) assessment of dense sucrose fractions containing *Cyanobium* Rubisco, CsoS1A and CsoS2 revealed rod-like carboxysome-like sub-structures, consistent with carboxysome structures that can form in the absence of vertex proteins (Cai et al. 2009; Long et al. 2018). These structures contained sub-components reminiscent of Rubisco holoenzymes, supporting the conclusion that Rubisco was successfully incorporated into simplified carboxysomes (Figs. 4a, S4a, S5a).

Analyses of sucrose gradient preparations of the *E. coli* cell line expressing wild type or mutant *T. elongatus* Rubisco with *Cyanobium* CsoS1A and CsoS2 show successful encapsulation but abnormal organisation of non-native cargo. Co-expression of the *Cyanobium* carboxysome components with wild type or mutant *T. elongatus* Rubisco yielded more free (unencapsulated) Rubisco (Figs. 4, S4). The composition of WT *T. elongatus* Rubisco-carboxysome preparations show a Rubisco peak that co-occurs with high proportions of CsoS1A and CsoS2, consistent with successful encapsulation (Figs. 4b, S4b, S5b). However, when fractions were visualised using TEM, the ordered packing within the carboxysome-like structures observed for *Cyanobium* Rubisco was not consistently seen with the non-native *T. elongatus* Rubisco (Figs. 4b, S5b). Similar observations were made for sucrose gradient purified carboxysome structures from *E. coli* expressing the *T. elongatus* Rubisco RbcL R72F mutant with *Cyanobium* CsoS1A and CsoS2 (Figs. 4c, S4c, S5c).

**Fig. 5:**
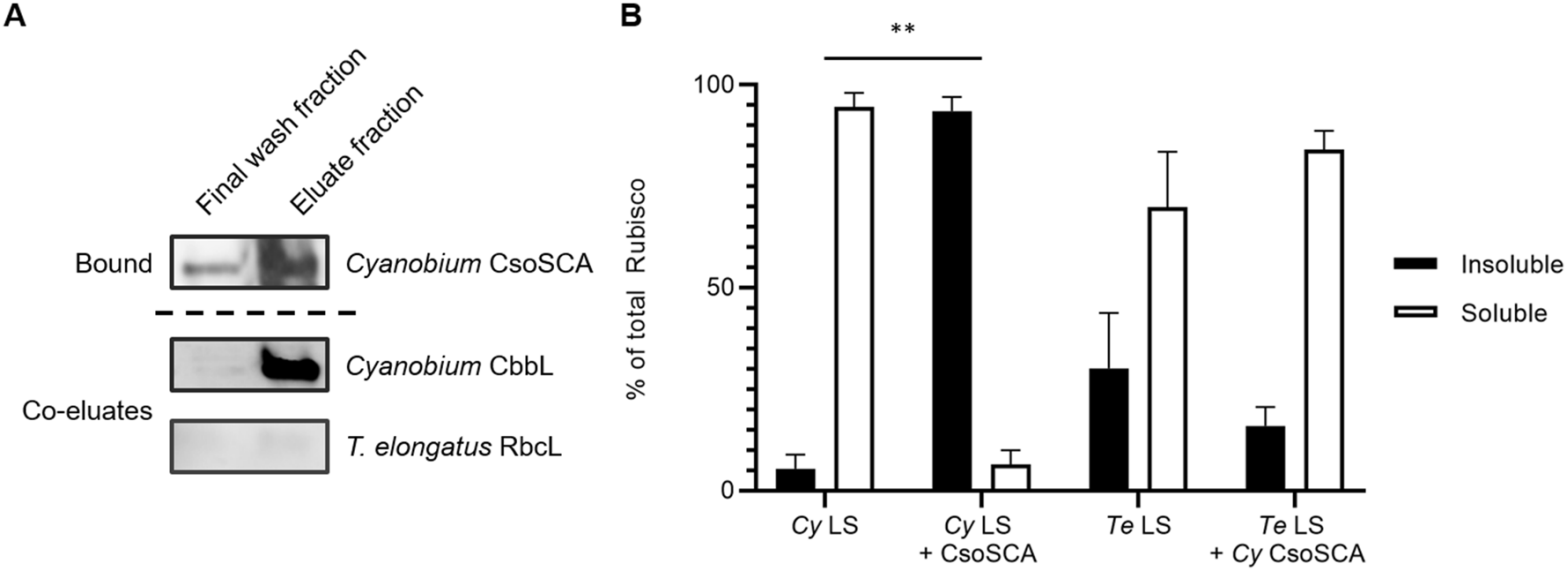
*Cyanobium* CsoSCA only readily interacts with its cognate Rubisco but not with *T. elongatus* Rubisco. **a** Western blots of eluted proteins from IMAC ‘bait’ (*Cyanobium* CsoSCA) and ‘prey’ (either *Cyanobium* or *T. elongatus* Rubisco separately) binding assays. Only *Cyanobium* CbbL was detected in the eluate fraction, whereas no band was observed for *T. elongatus* RbcL. **b** Solubility profiles from spin-down assays for *Cyanobium* (Cy) and *T. elongatus* (Te) Rubisco, on their own and when mixed with *Cyanobium* (CA) CsoSCA. *Cyanobium* Rubisco becomes readily pelletable when mixed and ‘spun-down’ with its cognate CsoSCA (one-way ANOVA; **p < 0.01). However, *T. elongatus* Rubisco does not share the same solubility properties when mixed and ‘spun-down’ with *Cyanobium* CsoSCA

### *Cyanobium* carbonic anhydrase in the context of a hybrid carboxysome

While Rubisco and CsoS2 are major components of the α-carboxysome lumen, carbonic anhydrase (CsoSCA) is also present and is critical for carboxysome functionality (Badger and Price 1994). Blikstad et al. (2021) recently detailed the Form IA Rubisco:CsoSCA interaction responsible for CsoSCA encapsulation in the *Halothiobacillus neapolitanus* α-carboxysome system, in which the CsoSCA N-terminal intrinsically disordered region (IDR) binds to the Rubisco holoenzyme. While the key residues for the Rubisco-CsoSCA interaction were highly conserved across the Form IA Rubiscos, only D99 and F356 (*H. neapolitanus* CbbL sequence numbering) were conserved in the Form IB Rubiscos (Figure S7). Moreover, the conserved arginine at Y72 would be highly disruptive of the observed CsoSCA-CbbL interaction (Blikstad et al. 2021).

To assess if *Cyanobium* CsoSCA can interact with Form IA and Form IB Rubisco, *Cyanobium* CsoSCA was bound to an IMAC column and separately challenged with either the *Cyanobium* Form IA Rubisco or the *T. elongatus* Form IB Rubisco. Only *Cyanobium* Form IA Rubisco bound to CsoSCA columns, revealing *Cyanobium* Rubisco and CsoSCA interaction is highly specific to the system, moreso than the CsoS2 interaction despite binding to a similar site on the Rubisco holoenzyme (Fig. 5a).

Rubisco:CsoSCA binding was further assessed with an independent co-precipitation assay. As similar types of disordered scaffold:Rubisco interactions have been demonstrated to phase-separate in this system (Wunder et al. 2018; Wang et al. 2019; Flecken et al. 2020; He et al. 2020; Oltrogge et al. 2020; Zang et al. 2021), we hypothesised that CsoSCA binding partners would co-aggregate in a manner reminiscent of phase-separated droplets. These assemblies may then be extracted from solution as insoluble protein. Alone, *Cyanobium* Form IA Rubisco and *T. elongatus* Form IB Rubisco were highly soluble when in solution (Figs. 5b, S6). *Cyanobium* Rubisco became predominately insoluble when mixed with *Cyanobium* CsoSCA, consistent with the known interaction partners forming insoluble assemblies in solution (Blikstad et al. 2021, Figs. 5b, S6, Table S2). Comparatively, *T. elongatus* Rubisco remained predominately soluble when mixed with *Cyanobium* CsoSCA, indicating these two proteins do not interact in solution in this manner (Figs. 5b, S6, Table S2).

## Discussion

### Characterisation of CsoS2-compatible *T. elongatus* Rubisco mutants

The heterologous expression of Rubisco has been widely examined (Whitney et al. 2015; Aigner et al. 2017; Lin et al. 2020) and these studies have collectively elucidated mechanistic actions for chaperones (Hauser et al. 2015) and functions for specific amino acid residues (Knight et al. 1990; Genkov and Spreitzer 2009; Wang et al. 2011). In this study, we rationally designed *T. elongatus* Form IB Rubisco mutants that may have increased affinity for CsoS2 as a foundational step for hybrid carboxysome construction. Here, we found the *T. elongatus* Form IB Rubisco mutants modified to interact with the α-carboxysome protein CsoS2, still retained their ability to undergo chaperone-assisted oligomerisation while their catalytic rates were also unaffected (Figs. 2, 3, S2). We therefore conclude that the mutated Rubisco amino acid residues in question, RbcL F72 and RbcS Y96, have no significant implications for Rubisco holoenzyme structure or activity and do not impede the enzyme’s chaperone interactions, an observation consistent with previous structural Rubisco analyses (Saschenbrecker et al. 2007; Xia et al. 2020; Li et al. 2022).

While the single *T. elongatus* Rubisco mutants were largely unaffected (RbcL R72F and RbcS I96Y), the double *T. elongatus* Rubisco mutant (RbcL R72F/RbcS I96Y) could not readily self-assemble and exhibited an exacerbated Rubisco chaperone requirement (Figs. 3, S2). This self-assembly deficiency was only restored in the presence of at least one Rubisco chaperone (Figs. 3, S2). The exact cause of this is unclear, however, it is possible that the charge difference conferred by these mutations do not favour Rubisco self-assembly with *E. coli* chaperones leading to an increased dependence on the heterologously expressed Rubisco chaperones. While the cyanobacterial Rubisco chaperones RbcX and Raf1 have been previously demonstrated to be important for native holoenzyme assembly (Huang et al. 2019, 2020), we further demonstrate that the optimal folding of cyanobacterial Rubisco mutants may only be achieved by the co-expression of cognate chaperones.

One justification for introducing a Form IB Rubisco into a simple α-carboxysome was to introduce the characteristically faster kinetics of Form IB Rubiscos. The addition of a His-tag to the C-terminus of *T. elongatus* Rubisco significantly altered the holoenzyme’s catalytic rate, but the introduction of further mutations did not significantly affect the catalytic rate (Table 1). Here we present single point analysis of Rubisco carboxylation turnover as a means for simple comparison between WT and mutant Rubisco function. Single-point enzyme activities generally underestimate true maximum turnover rates due to the inability to capture truly saturating rates of catalysis. Notably, Zhou and Whitney (2019) reported *T. elongatus* Rubisco to have a *k*_cat_ value of 6.6 s^−1^, higher than our derived value of 4.4 s^−1^ (Table 1). While this particular Form IB Rubisco is not an especially fast carboxysomal enzyme, its sequence identity in relation to CcmM and CsoS2 interaction sites is identical to that of the fastest reported carboxysomal enzyme from *S. elongatus* PCC7942 (Whitehead et al. 2014). Here, we demonstrate the holoenzyme’s activity is not significantly affected by targeted mutations and therefore it is possible that faster Form IB Rubisco homologues could be similarly mutated for α-carboxysome encapsulation, without compromising their catalytic turnover rate.

### Unmodified *T. elongatus *Rubisco can interact with *Cyanobium* CsoS2

The current model of α-carboxysome biogenesis describes a process of concurrent assembly in which nascent shells form alongside internal cargo structure (Oltrogge et al. 2020). Central to this process is the initial formation of Rubisco:CsoS2 interactions, believed to involve the formation of condensates, leading to high densities of CsoS2 that in turn, interacts with α-carboxysomal shell proteins (Oltrogge et al. 2020). Building on the recent characterisation of Rubisco:CsoS2 interaction by Oltrogge et al. (2020), we demonstrate a route for non-native Rubisco access to α-carboxysome interactions. Our results highlight that the existing conserved residues between Form IA and Form IB Rubiscos are sufficient for interactions with CsoS2. Wild-type and mutant *T. elongatus* Form IB Rubiscos both appear to colocalise with α-carboxysome components, indicative of successful encapsulation (Fig. 4). Therefore, there is no need to ensure all the CsoS2 binding residues are transferred from Form IA Rubiscos to Form IB Rubiscos to enable heterologous cargo recruitment (Fig. 4).

A key difference between the Rubisco-disordered scaffold binding interfaces of α- and β-carboxysomes is the presence of cation-π interactions in the CsoS2:Form IA Rubisco interface (Wang et al. 2019; Oltrogge et al. 2020). Indeed, Oltrogge et al. (2020) found that the Y72R mutation of *H. neapolitanus* Form IA Rubisco (in essence, the opposing mutation to that described here introducing the conserved Form IB Rubisco arginine residue into a Form IA context) inhibited interactions with its cognate CsoS2. The fact that introducing this aromatic residue in the Form IB Rubisco promotes Rubisco:CsoS2 binding reinforces the importance of such interactions in the α-carboxysome and further underlines the critical distinction between the α- and the β-carboxysome systems, likely contributing to specificity (Oltrogge et al. 2020; Blikstad et al. 2021). Whether CcmM could facilitate such a hybrid interaction (i.e., Form IA Rubisco:CcmM) is an interesting question for future research and would further deepen our understanding of Rubisco interactions with disordered scaffolds in each system.

The internal structure of the α-carboxysome is characterised by tight, ordered Rubisco packaging (Metskas et al. 2022; Ni et al. 2022). While this was observed in the simplified carboxysomes when nascent *Cyanobium* Rubisco was present (Figs. 4a, S5a), this was not consistently seen from the co-expression of *T. elongatus* Rubisco with *Cyanobium* CsoS1A and CsoS2 (Figs. 4b, c, S5b, S5c), which may point to the absence of correct Rubisco:carboxysome-shell interactions. To date, there is limited evidence of Form IA Rubisco interacting with CsoS1 (Gonzales et al. 2005) however the exact interaction remains unresolved.

These observations may also speak to the differences between the Rubisco:scaffold interface properties of each system. Notably, CsoS2 binds simultaneously to eight available sites on the Form IA Rubisco holoenzyme, whereas CcmM occludes neighbouring sites leading to four Form IB Rubisco holoenzyme interfaces (Wang et al. 2019; Oltrogge et al. 2020). Consequently, the CsoS2 interaction appears to have greater multivalency and tighter binding, potentially leading to very different branching architecture relative to the β-carboxysome counterpart. *T. elongatus* Rubisco maybe optimised for these lower valency CcmM internal cargo matrices, leading to the aggregate-like structure observed in the α-carboxysome context (Figs. 4b, b, S5b, S5c). Additionally, cation-π contacts present in the Form IA Rubisco:CsoS2 interaction, have been highlighted as key drivers of phase separation (Wang et al. 2018; Oltrogge et al. 2020). Their absence from the Form 1B Rubisco:CsoS2 interaction may lead to more rigidity in the matrix, precluding the diffusion of Rubisco within hybrid carboxysomes. Broadly, this finding highlights how little we know about what determines internal carboxysome matrix properties.

### Future considerations for the carbonic anhydrase in a hybrid carboxysome

While we have focussed on the Rubisco:CsoS2 interaction, recent work has also elucidated the Rubisco:CsoSCA interaction (Blikstad et al. 2021). CsoSCA is believed to compete with CsoS2 for Rubisco, binding at nearly the same location on the holoenzyme but employing divergent binding modes (Blikstad et al. 2021). The CsoSCA disordered region is buried much deeper within the CbbL subunits and interacts primarily through hydrogen bonds and a seemingly ordered network of water molecules. While Y/F72 is implicated in CsoSCA binding, the orientation and mode of this interaction is notably different to that which mediates CsoS2 interactions (Fig. 5; Oltrogge et al. 2020; Blikstad et al. 2021). It is also worth noting the unusually high proline content of the CsoSCA N-terminal disordered region (Levitt 1981; Blikstad et al. 2021). This likely restricts the conformational dynamisim of the peptide, thus severely limiting its capacity to adopt as many alternate conformations as CsoS2, precluding orientations that enable Form IB Rubisco interactions. Future attempts to engineer an α-carboxysome-compatible Form IB Rubisco will also need to verify that the Rubisco:CsoSCA interaction is maintained to ensure proper carboxysome functionality as previously demonstrated and modelled (Price and Badger 1989; Fukuzawa et al. 1992; Price et al. 1992; Yu et al. 1992; So et al. 2002; Dou et al. 2008; Long et al. 2021).

In conclusion, mutations made to engineer *T. elongatus* Rubisco to be more CsoS2-compatible did not appear to alter its chaperone or kinetic characteristics. However, *Cyanobium* CsoS2 could interact with unmodified *T. elongatus* Rubisco under appropriate salt concentrations. While *T. elongatus* Rubisco was encapsulated within an α-carboxysome-like structure, the overall appearance of resultant structures suggested further unresolved α-carboxysomal interactions, with one example being the Rubisco:CsoS1A interaction interface. A complete understanding of the α-carboxysome structural interactions will assist in future attempts to successfully incorporate a foreign, faster Rubisco to generate a hybrid carboxysome.

## Methods

### Genetic constructs

To examine the effects of *T. elongatus* Rubisco chaperones RbcX and Raf1 on folding of modified Rubisco, genes for *T. elongatus* BP-1 Rubisco (*rbcL* and *rbcS*), RbcX (*rbcX*) and Raf1 (*raf1*) were codon optimised for *E. coli,* synthesised as an operon by Genewiz (USA) and delivered in the pUC57 plasmid vector (Figure S1). It should also be noted that *rbcL* was synthesized with a 6 × His-tag (HHHHHH), *rbcX* was synthesised with an HA-tag (YPYDVPDYA) and *raf1* was synthesised with a myc-tag (EQKLISEEDL) to facilitate immunoblot detection and immobilised metal affinity chromatography (IMAC). *Eco*RI and *Hind*III restriction cut sites flanked the operon to fit the operon into the pUC57 plasmid, while *Nco*I and *Xba*I restriction cut sites were added to facilitate the transfer of the operon into the pSE2.1 expression vector (Figure S1). Further restriction sites were included in this operon to facilitate simple gene excision.

The *T. elongatus* BP-1 *rbcL* gene was then replaced with the mutant variant by digestion with *Nco*I and *Bam*HI, while the *T. elongatus* BP-1 *rbcS* gene was replaced by digestion with *Bam*HI and *Spe*I. Genes for *Cyanobium csoS1A* and *csoS2* were flanked with *Stu1* restriction cut sites which were used to introduced these genes into the operon containing genes for *T. elongatus* BP-1 Rubisco (*rbcL* and *rbcS*), RbcX (*rbcX*) and Raf1 (*raf1*) (Figure S1).

### Mutagenesis PCR

Primers were designed to flank the gene of interest and overlap the mutation site, and were synthesised by Sigma-Aldrich (USA) (Table S1). The first PCR cycle utilised the Herculase II Fusion DNA polymerase (Agilent, USA), with the proprietary protocol followed and an annealing temperature of 67 °C used for all reactions.

Resultant forward and reverse fragments were separated on a 1.5% TAE (40 mM Tris base, 20 mM acetic acid, 1 mM EDTA) agarose gel and purified using the Wizard® SV Gel and PCR Clean-Up System (Promega, USA). Purified forward and reverse fragments were then recombined in a second PCR cycle using the TaqTi polymerase (Fisher Biotec, USA) initially in the absence of flanking primers (*rbcL_F*/*rbcL_R*/*rbcS_F*/*rbcS_R*; Table S1).

### Escherichia* coli* protein expression assays

Overnight *E. coli* cultures (5 mL) grown at 37 °C and induced with 50 μM IPTG were normalised to the equivalent of 1 mL of OD_600_ = 0.6. Cells were pelleted, resuspended in gel loading buffer (Laemmli Sample Buffer [Bio-Rad, USA], 50 mM DTT), boiled at 95 °C and clarified by centrifugation. Proteins from the crude lysate were then separated at 210 V for 35 min in denaturing buffer (1% [*w*/*v*] SDS, 25 mM Tris, 50 mM glycine) on a 4–20% SDS-PAGE gel (BioRad, USA). Separated proteins were transferred from onto an Immobilon-P PVDF membrane (Merck, USA) and blocked in TBS-T buffer (50 mM Tris, 150 mM NaCl, 0.1% [*v*/*v*] Tween-20 [Sigma, USA]) containing 2.5% skim milk powder. Blocked membranes were probed with polyclonal antibodies raised against tobacco Rubisco (1:5000 dilution, gifted by S.M. Whitney), *Cyanobium* CsoS1A (1:5000 dilution, prepared by Genscript, USA) and *Cyanobium* CsoS2 (1:5000 dilution, prepared by Genscript, USA) and monoclonal antibodies raised against myc (1:5000 dilution, prepared by Sigma, USA) and HA (1:5000 dilution, prepared by Sigma, USA). For polyclonal antibodies, the probe signal was detected with alkaline phosphatase-conjugate anti-rabbit secondary antibody (1:5000 dilution, prepared by Sigma, USA), while for monoclonal antibodies, the probe signal was detected with alkaline phosphatase-conjugate anti-mouse secondary antibody (1:5000 dilution, prepared by Sigma, USA). Probed blots were visualised using the Attophos Substrate Kit (Promega, USA) and the BioRad ChemiDoc XRS + system (BioRad, USA).

### Native gel analysis of assembled Rubisco from *E. coli*

A 5 mL starter *E. coli* culture was grown for 8 h at 37 °C, from which the equivalent of 1 mL of OD_600_ = 0.1 was used to inoculate an overnight 40 mL culture, induced with 50 μM IPTG and grown at 37 °C. The equivalent to 40 mL of OD_600_ = 0.6 was collected by centrifugation (6000×*g* for 10 min), taken up in 5 mL TE (10 mM Tris–HCl, pH 8.0, 1 mM EDTA) buffer with bacterial protease inhibitor cocktail (Sigma, USA) and lysed with three passes of the Emulsiflex (Avestin, USA) set at 60psi. Lysates were clarified by centrifugation (10,000×*g* for 10 min) and proteins from the resultant supernatant were separated at 150 V for 150 min in non-denaturing buffer (25 mM Tris, 50 mM glycine). Proteins were fixed with protein fixing solution (50% methanol, 10% acetic acid, 40% H_2_O; *v*/*v*/*v*) for no longer than 30 min, washed with H_2_O, incubated with GelCode Blue Safe Stain (ThermoFisher, USA), washed with H_2_O and visualised with the BioRad ChemiDoc XRS + system (BioRad, USA). Bands were quantified using the Image Lab software package (Bio-Rad, ver 6.1.0 build 7).

### Rubisco kinetic activity assays

Rubisco catalytic turnover rates were derived from single point Rubisco activity assays (using the protocol described by Whitney and Sharwood (2007) and CABP binding assays (as described by Whitney and Sharwood (2007)). Briefly, Rubisco activity assays utilized the conversion of RuBP into acid-stable PGA and were performed at 25 °C with a 5 min activation time and a 5 min reaction time, while Rubisco active sites were quantified with CABP binding assays. Both single point Rubisco activity assays and CABP binding assays were performed in triplicate.

### Sucrose gradient carboxysome purification and visualization

Carboxysome-like structures were purified from *E. coli* using the protocol described by So et al. (2004). A 5 mL starter *E. coli* cultures expressing only *Cyanobium* PCC7001 Rubisco, CsoS1A and CsoS2, and *T. elongatus* BP-1 Rubisco co-expressed with *Cyanobium* PCC7001 CsoS1A and CsoS2 were grown overnight at 37 °C from which 1 mL was taken to inoculate a 40 mL culture allowed to grow for 8 h at 37 °C. The 40 mL intermediary culture was then used to inoculate a 1L culture grown overnight at 23 °C and induced with 50 μM IPTG. The following day, cultures reached an approximate OD_600_ = 1, were harvested by centrifugation (6000×*g* for 10 min) and resuspended in 5 mL TEMB buffer. Resuspended cell solutions were lysed with three passes of the Emulsiflex (Avestin, USA) set at 60psi, diluted with TEMB and 2.5% Triton X-100 (Sigma, USA) to a final volume of 35 mL and left rotating at 23 °C for at least an hour. Cell debris was removed by centrifugation (3000×*g* for 5 min) and the resultant supernatant was subjected to a hard centrifugation step to generate a carboxysome-enriched pellet (45,000×*g* for 20 min). The carboxysome-enriched pellet was gently resuspended with 1 mL TEMB aided by a paintbrush and subjected to a further gentle clarification step (3000×*g* for 1 min). 1 mL of the resultant supernatant was transferred to a TEMB linear sucrose gradient (5–60% sucrose) and centrifuged (100,000×*g* for 1 h). 1 mL fractions were collected from the sucrose gradient and proteins in each fraction were separated on a SDS-PAGE gel, transferred on a PVDF membrane and probed with antibodies raised against Rubisco, CsoS1A and CsoS2. Aliquots of sucrose gradient fractions that contained the greatest amount of Rubisco, CsoS1A and CsoS2 were negatively stained with 2% uranyl acetate and visualised using the Hitachi 7100 TEM.

### CsoSCA IMAC binding assays

Single transformed *E. coli* (strain BL21 DE3) with desired expression cassettes were grown in 5 mL LB with appropriate antibiotics for 8 h at 37 °C. These were then used to inoculate 400 mL LB and grown overnight at 37 °C. The next day overnight cultures were diluted 1:5 in fresh LB to generate 2L batch cultures. Clarified supernatant was collected and passed through 0.45 μm syringe filter (Merck, USA). Supernatants of His-tagged proteins were applied to pre-washed columns with 0.25 mL bed volume of IMAC Ni^2+^ Charged Resin (Bio-Rad, USA). Protein-bound columns were washed with 10 mL of Standard Binding Buffer (50 mM Tris [pH 7.8], 300 mM NaCl, 25 mM Imidazole). Lysates containing untagged proteins of interest were then passed over the column. Columns were then washed with 30 mL of Standard Binding Buffer. Proteins were eluted with 3 mL High-Salt Elution Buffer (50 mM Tris [pH 7.8], 600 mM NaCl, 500 mM Imidazole). Protein samples were taken throughout the extraction and binding assay for SDS-PAGE and western blot analysis as described. To detect *Cyanobium* CsoSCA, membranes were probed with polyclonal antibodies raised against *Cyanobium* CsoSCA (1:5000 dilution, prepared by Genscript, USA).

### Rubisco-CsoSCA spin-down assays

*E. coli* (strain BL21 DE3) cell lines expressing proteins of interest were grown in 5 mL LB with appropriate antibiotics for 8 h at 37 °C. These were then used to inoculate 400 mL LB and grown overnight at 37 °C. These were diluted 1:5 in fresh LB to generate 2L batch cultures, were grown for 1 h at 37 °C and then induced with 100 μM IPTG and grown for 3.5 h at 28 °C. Cells were harvested by centrifugation at 6000×*g* for 15 min, lysed with three passes of the Emulsiflex (Avestin, USA) and clarified by centrifugation (10,000 × *g*, 15 min, 4 °C). Cells expressing CsoSCA64 were broken in 5 mL High-Salt Extraction Buffer (50 mM Tris [pH 7.8], 600 mM NaCl) while all other lines were extracted in 5 mL Standard Extraction Buffer (50 mM Tris [pH 7.8], 300 mM NaCl). Equivalent amounts of each lysate sample were diluted to 4.5 mL of 150 mM NaCl. Lysates expressing proteins of interest were combined with each other or buffer controls to a final volume of 0.5 mL and incubated on ice for 30 min. Aggregates were collected by centrifugation at 10,000×*g* for 5 min. Soluble fractions were collected after centrifugation. Pellets were then resuspended to 0.5 mL in fresh Extraction buffer of the appropriate NaCl concentration and samples taken. All samples were analysed through SDS- PAGE and western blots probed for proteins of interest in each sample. Band intensities of each sample were compared and quantified using the Image Lab software package (Bio-Rad, ver 6.1.0 build 7).

## Supplementary Information

Below is the link to the electronic supplementary material.

Supplementary file1 (DOCX 3093 kb)
