# Supplementary material for: Towards engineering a hybrid carboxysome
Source: Photosynth Res. 2023 Mar 9;156(2):265–77. doi: 10.1007/s11120-023-01009-x (PMC10154267; doi:10.1007/s11120-023-01009-x)
Supplement: Supplementary file 1 — Supplementary file1 (DOCX 3093 kb) [file 11120_2023_1009_MOESM1_ESM.docx]

**
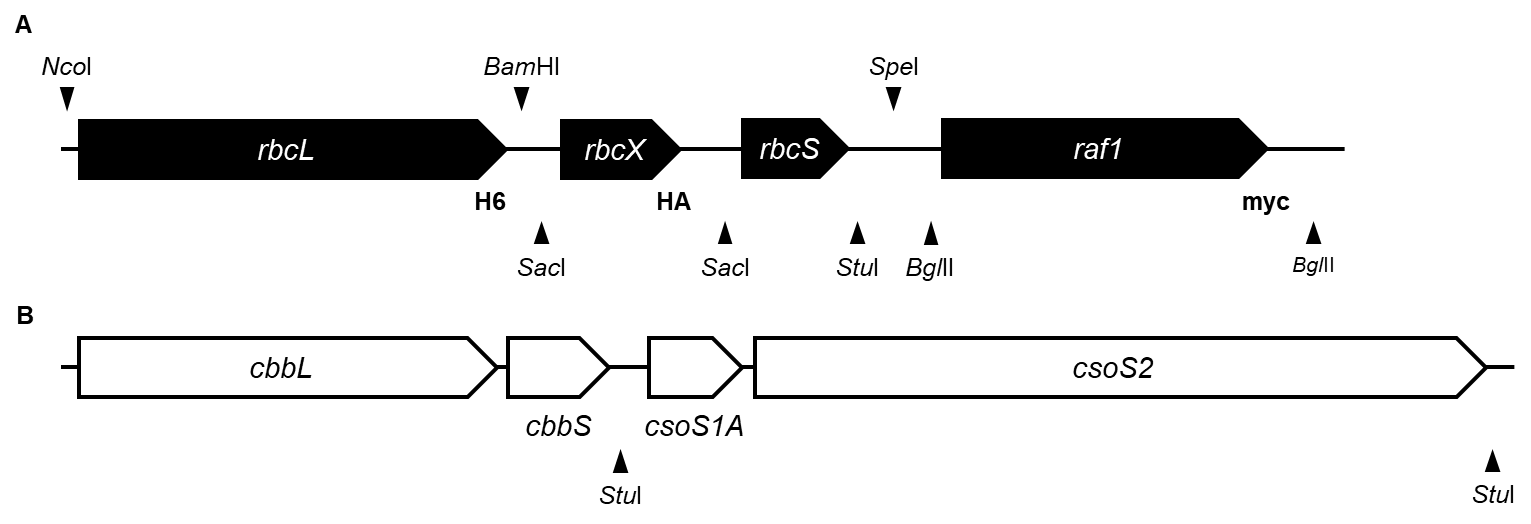
Supplemental Figures and Tables**

**Figure S1. Schematic of constructs used to express and verify the chaperonal requirements for mutant *T. elongatus* Rubiscos (A) and construct a simplified *Cyanobium* carboxysome (B).** To express *T. elongatus* Rubisco, Rubisco genes (*rbcL* and *rbcS*) were co-expressed with genes for Rubisco chaperones RbcX and Raf1 (A). Here, *rbcL* was synthesized with a 6 × His-tag (HHHHHH), *rbcX* was synthesised with a HA-tag (YPYDVPDYA) and *raf1* was synthesised with a myc-tag (EQKLISEEDL) to facilitate immunoblot detection. Additional restriction sites were included to facilitate simple gene excision, *rbcL* is removed by digestion with *Nco*I and *Bam*HI and the *rbcX-rbcS* fragment is removed by digestion with *Bam*HI and *Spe*I. *Sac*I removes *rbcX* and digestion with *Bgl*II removes *raf1*. To construct a simplified *Cyanobium* carboxysome, genes for Rubisco (*cbbL* and *cbbS*) were co-expressed with genes for the carboxysome protein CsoS1A and CsoS2 (B). *Stu*I cut sites that flank the *csoS1A*-*csoS2* fragment can then be used to transfer this fragment into the *T. elongatus* Rubisco expression cassette (A).

| Primer name | Sequence (5’ to 3’) |
| --- | --- |
| *rbcL_F* | AACCATGGCCTATACGCAATCCAAATCCCA |
| *rbcL_R* | TCGGATCCACCAACTAATGGTGATGGTGA |
| L R72F F | GCTGACCGACTTGGAT*TT*CTACAAAGGC |
| L R72F R | GCCTTTGTAG*AA*ATCCAAGTCGGTCAGC |
| *rbcS_F* | TGGATCCGAGCTCGGGTTTAATCGGAGAAG |
| *rbcS_R* | ACACTAGTAGGCCTTAATAGCGATAACCGCTG |
| S I96Y F | GTTGCCTTTGACAACT*AC*AAACAGTGCCAAG |
| S I96Y R | CTTGGCACTGTTT*GT*AGTTGTCAAAGGCAAC |

**Table S1. Primers required to introduce mutations in both *T. elongatus* BP-1 *rbcL* and *rbcS*. All primers were synthesised by Sigma-Aldrich (USA).** Unique restriction sites are underlined, while introduced mutation regions are italicised.


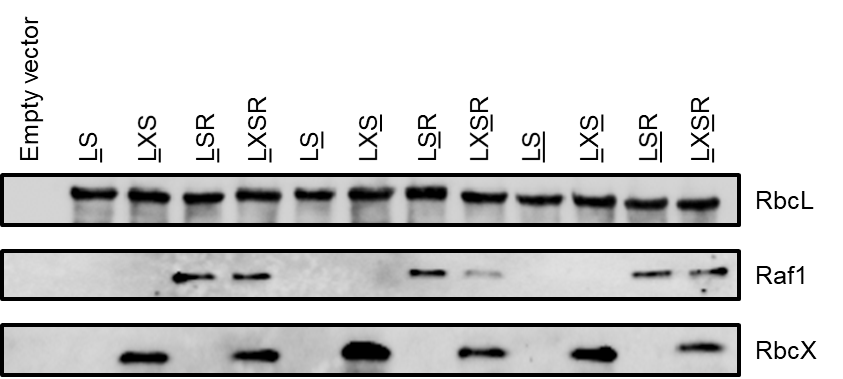


**Figure S2. Expression of engineered versions of *T. elongatus* Rubisco components and chaperones Raf1 and RbcX in *E. coli*.** Western blots of proteins from clarified lysates of *E. coli* cells expressing an empty pSE2.1 vector control, *T. elongatus* Rubisco, RbcX and Raf1 (LXSR), and mutated *T. elongatus* Rubisco (RbcL R72F denoted as L, RbcS I96Y denoted as S and RbcL R72F/RbcS I96Y denoted as LS) without either Rubisco chaperone (LS), with RbcX (LXS) or Raf1 (LSR) alone, and with both RbcX and Raf1 (LXSR). All *T. elongatus* proteins and mutant variants were expressed in *E. coli*.


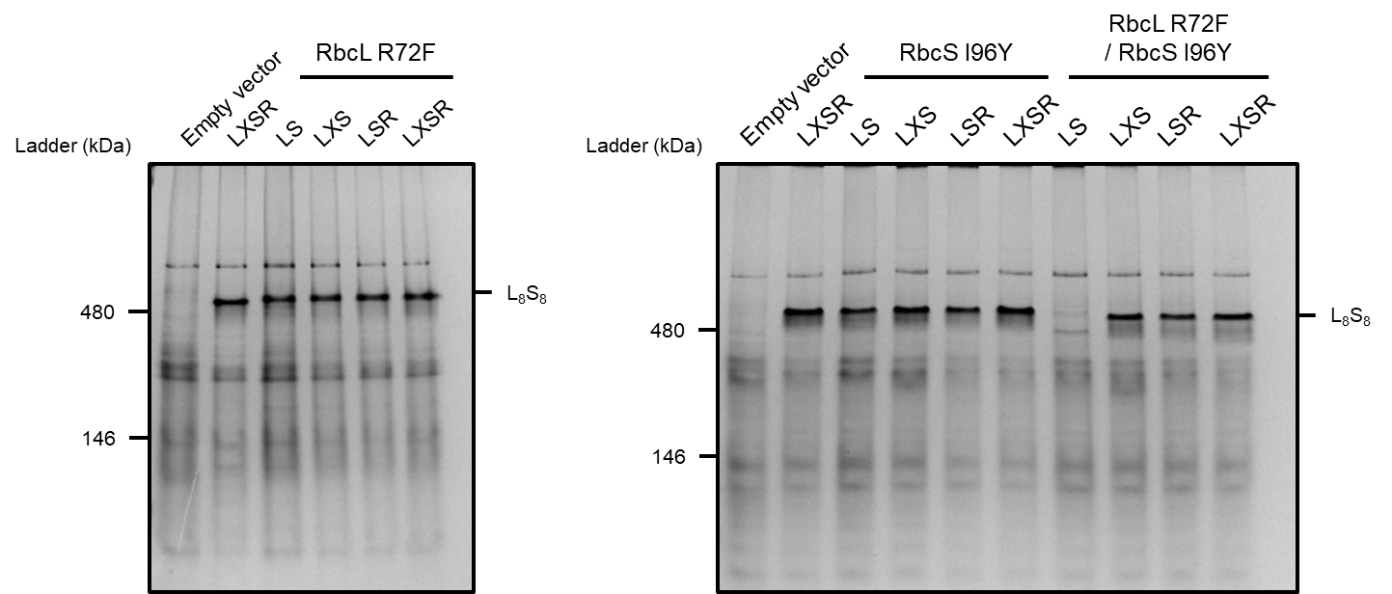


**Figure S3. Mutated *T. elongatus* Rubisco can still assemble into a holoenzyme and undergo chaperone-assisted folding.** Representative Coomassie stained native gel of soluble proteins from clarified *E. coli* cells expressing an empty pSE2.1 vector control, *T. elongatus* Rubisco with RbcX and Raf1 (LXSR), *T. elongatus* Rubisco mutant variants (RbcL R72F, RbcS Y69I and RbcL R72F/RbcS Y69I) co-expressed alone (LS), with RbcX (LXS) or Raf1 (LSR), or with both RbcX and Raf1 (LXSR). Mutations introduced into *T. elongatus* to make it CsoS2 compatible did not prevent holoenzyme assembly or chaperone-assisted folding by RbcX and Raf1.


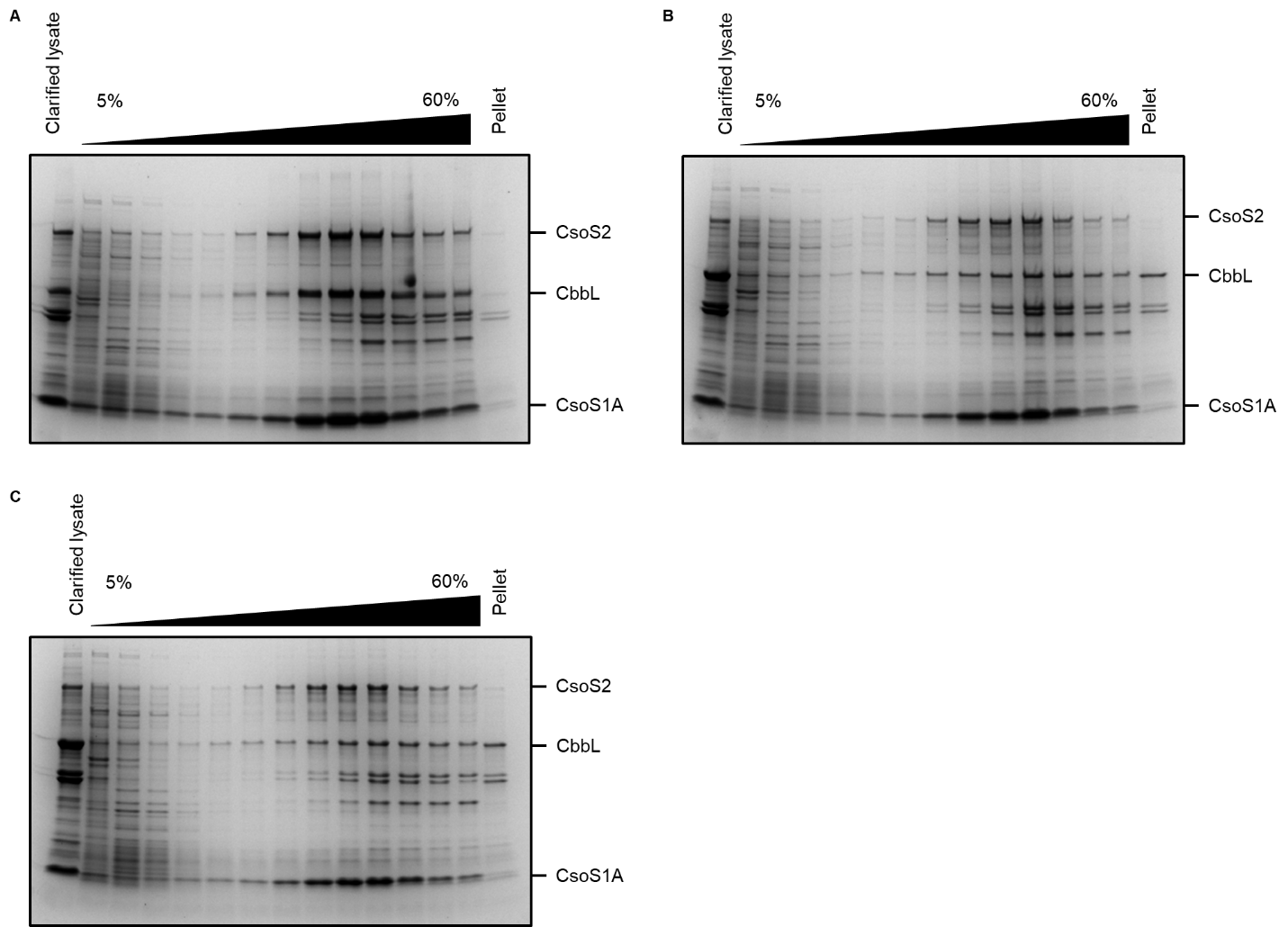


**Figure S4. Co-expression of *Cyanobium* CsoS1A and CsoS2 with *Cyanobium* Rubisco (A), wild-type (B) and mutant (C) *T. elongatus* Rubisco produces carboxysome-like structures that migrate down a TEMB sucrose (5-60%) gradient.** Proteins from the clarified lysate (diluted 1:12), 1 mL sucrose gradient fractions and the pellet (diluted 1:100) were separated on a SDS-PAGE gel, fixed and stained with Coomassie stain. The fraction that contained the most Rubisco, CsoS1A and CsoS2 was then visualised with TEM.


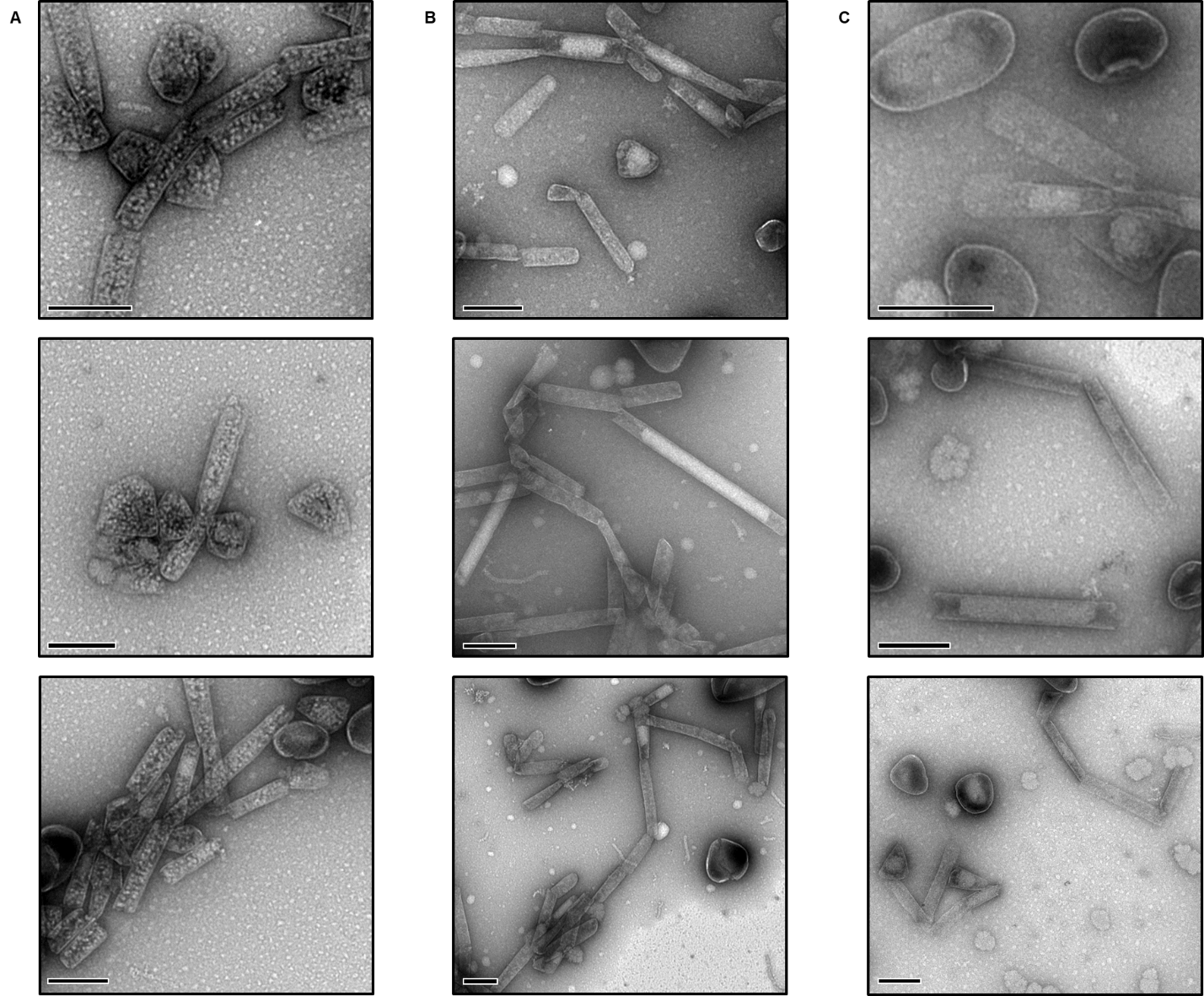


**Figure S5. Representative electron micrographs of sucrose-gradient purified carboxysome structures produced from the co-expression of *Cyanobium* CsoS1A, CsoS2 (A) and *Cyanobium* PCC7001 Rubisco, wild-type (B) or mutant (C) *T. elongatus* Rubisco.** Fractions with the greatest amount of Rubisco, CsoS1A and CsoS2 as determined using western blots, were visualised with TEM. Simplified *Cyanobium* carboxysomes were observed from the co-expression of *Cyanobium* Rubisco, CsoS1A and CsoS2 (A). Co-expression of wild-type (B) and mutant (C) *T. elongatus* BP-1 Rubisco with *Cyanobium* CsoS1A and CsoS2 produced rod-like carboxysome structures with abnormal internal structure.


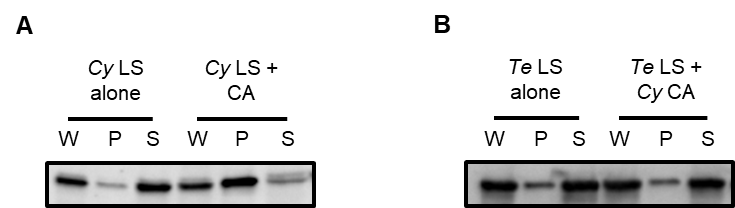


**Figure S6. Representative solubility profiles for *Cyanobium* and *T. elongatus* Rubisco (LS) when mixed with *Cyanobium* carbonic anhydrase (CA) to quantify the effects of spin-down assays.** *E. coli* lysates containing *Cyanobium* LS, CA or *T. elongatus* LS were mixed in 150mM NaCl and left on ice for 30 minutes. These mixed solutions (W) were pelleted by centrifugation to give an insoluble (P) and soluble (S) fraction. Proteins from each fraction were separated on an SDS-PAGE gel, transferred onto a PVDF membrane and probed with polyclonal antibodies raised against the tobacco Rubisco large subunit. Rubisco band densities from the insoluble and soluble fraction were then scaled, such that the sum of the insoluble band (P) and the soluble band (L) was equal to the band density in the whole cell lysate (W), with the raw data presented in Table S2. In isolation *Cyanobium* LS is predominately soluble, however it becomes readily pelletable when mixed with CA (A). Comparatively, *T. elongatus* LS remains soluble even in the presence of *Cyanobium* CA (B).

|  |  | **Band density volumes (AU)** | | | **Adjusted band volumes (% of W)** | | |
| --- | --- | --- | --- | --- | --- | --- | --- |
|  |  | Replicate 1 | Replicate 2 | Replicate 3 | Replicate 1 | Replicate 2 | Replicate 3 |
| ***Cy* LS alone** | **W** | 8,618,528.00 | 14,608,506.91 | 7,341,884.89 |  |  |  |
|  | **P** | 1,052,432.20 | 198,553.72 | 347,386.78 | 8.94 | 1.99 | 5.27 |
|  | **S** | 10,721,846.84 | 9,775,579.00 | 6,238,947.23 | 91.06 | 98.01 | 94.73 |
| ***Cy* LS + *Cy* CA** | **W** | 6,275,369.82 | 14,634,863.30 | 9,638,541.71 |  |  |  |
|  | **P** | 14,718,299.16 | 12,748,308.75 | 15,266,867.40 | 89.57 | 96.24 | 94.85 |
|  | **S** | 1,714,364.45 | 498,244.26 | 829,429.91 | 10.43 | 3.76 | 5.15 |
| ***Te* LS alone** | **W** | 13,174,351.46 | 18,663,714.36 | 18,225,428.00 |  |  |  |
|  | **P** | 2,689,923.46 | 5,917,660.83 | 5,773,678.10 | 19.81 | 24.90 | 45.61 |
|  | **S** | 10,885,519.33 | 17,844,181.24 | 6,885,714.43 | 80.19 | 75.10 | 54.39 |
| ***Te* LS + *Cy* CA** | **W** | 9,517,234.61 | 20,331,692.40 | 12,007,171.03 |  |  |  |
|  | **P** | 2,065,722.61 | 3,097,999.88 | 1,470,550.40 | 15.55 | 20.77 | 11.47 |
|  | **S** | 11,219,676.07 | 11,817,619.82 | 11,352,674.63 | 84.45 | 79.23 | 88.53 |

**Table S2. Raw data for band densities used for Rubisco and CA spin-down assays in the presence of 150mM NaCl.** Proteins from whole cell lysates (W), insoluble lysate fractions (P) and soluble lysate fractions (S) were separated using SDS-PAGE gels, transferred onto PVDF membranes, probed for Rubisco large subunit with resultant band densities calculated using ImageLab. Rubisco band densities from the insoluble and soluble fraction were scaled, such that the sum of the insoluble band (P) and the soluble band (S) was equal to the band density in the whole cell lysate (W). The proportion of each sample was calculated and plotted, shown in Figure 5 of the main text.


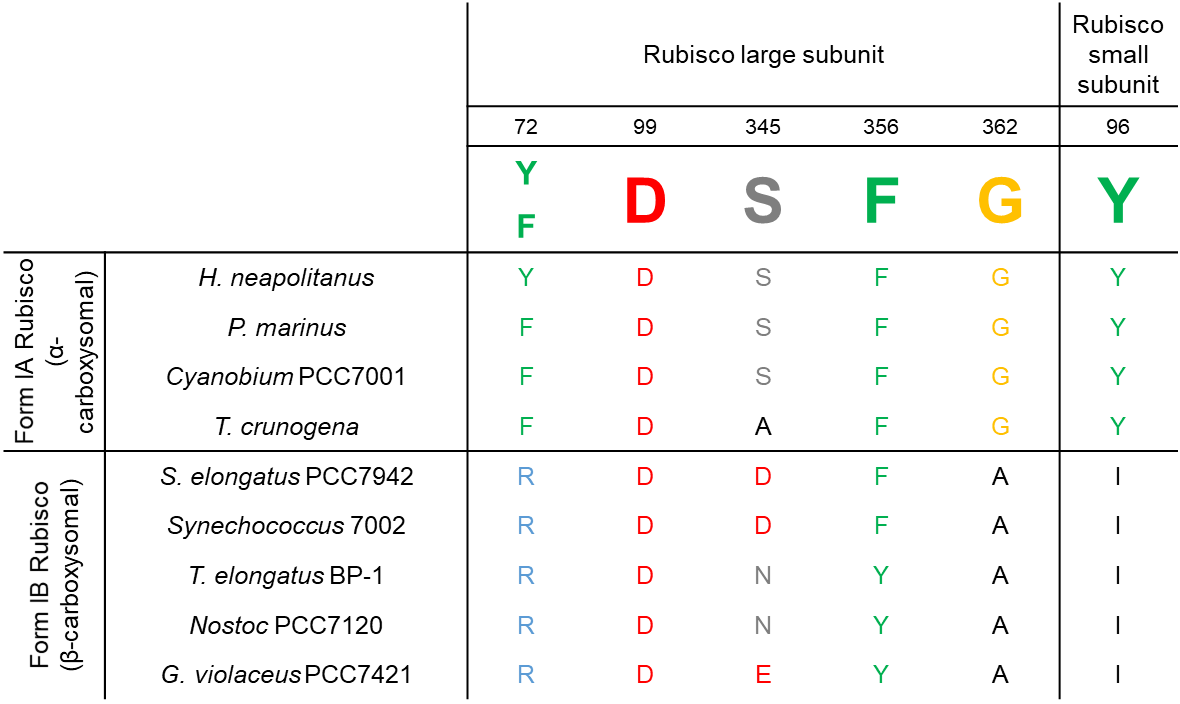


**Figure S7. Rubisco residues responsible for the Rubisco-CsoSCA interaction are not well conserved across both Form IA and IB Rubiscos.** Comparison of CsoSCA interacting amino acid residues across Form IA and Form IB Rubisco sequences with a Form IA Rubisco consensus sequence. Residues are numbered according to the *H. neapolitanus* Rubisco large and small subunit sequence. There is a high degree of conservation across the α-carboxysomal systems, however, only one residue (D99) is conserved in the β-carboxysomal system.
